# Molecular mechanisms of CYP-13 function in *C. elegans*: insights into conserved P450 pathways

**DOI:** 10.1007/s00204-025-04284-w

**Published:** 2026-01-14

**Authors:** Sharoen Yu Ming Lim

**Affiliations:** Department of Basic Medical Sciences, Faculty of Medicine and Health Sciences, Universiti Malaysia Sarawak, Jln Datuk Mohammad Musa, 94300 Kota Samarahan, Sarawak Malaysia

**Keywords:** *C. elegans*, *cyp-13*, *cyp-13A12*, EGL-9–HIF1–PUFA–eicosanoid pathway

## Abstract

Cytochrome P450 enzymes (CYPs) are central to metabolism and stress adaptation. In *Caenorhabditis elegans*, the CYP-13 family performs diverse and conserved functions beyond xenobiotic detoxification. *cyp-13* links lifespan regulation to the APP ortholog *apl-1* and the heterochronic factor *lin-14*, integrating with DAF-16/FOXO, HSF-1, and DAF-12 pathways. In apoptosis, *cyp-13* contributes to the degradosome complex with CPS-6/EndoG and WAH-1, facilitating DNA degradation. Several isoforms are inducible by aflatoxin B1 and PCB1254, underscoring roles in toxicant metabolism. Notably, *cyp-13A12* regulates behavioral responses to reoxygenation via the EGL-9–HIF-1–PUFA–eicosanoid pathway, paralleling mammalian ischemia–reperfusion responses. Epigenetic regulation adds another layer, as BRCA1/BARD1 homologs *brc-1* and *brd-1* repress distinct subsets of *cyp-13A* genes through H2A ubiquitylation. Collectively, CYP-13 emerges as a multifunctional hub linking developmental, apoptotic, metabolic, stress, and chromatin-level processes, with clear parallels to human CYPs, highlighting its translational relevance to aging, cancer, and toxicology.

## Introduction

Cytochrome P450 (P450s or CYPs) enzymes are one of the most versatile biocatalysts in nature. CYPs responsible for the oxidative metabolism of a broad range of endogenous and exogenous compounds (Iacopetta et al. 2023). Drug–drug interactions are major mechanisms caused by the inhibition and/or induction of CYP enzymes (Armani et al. 2024). This phenomenon is of profound clinical significance since CYP-mediated metabolism determines the pharmacokinetic fate of many therapeutic agents (Liu et al. 2022). CYPs are also involved in diseases such as alcoholic and non-alcoholic fatty liver disease (Jiang et al. 2024), inflammatory and immune responses in events of respiratory diseases e.g. COVID-19 (Lim et al. 2023a), neurodegenerative diseases (Durairaj and Liu 2025), and intestinal epithelial injury (Chen et al. 2022). Reducing CYPs activity represents a promising therapeutic strategy for certain diseases (Guengerich 2024). Pharmacological inhibition of CYP2E1 has been shown to attenuate oxidative liver injury in models of alcoholic fatty liver disease (Alshehade et al. 2022; Zhu et al. 2025), while selective suppression of CYPs involved in pro-carcinogen activation could reduce cancer risk (Jia et al. 2025). However, despite the identification of specific P450 isoforms as potential drug targets (Lim et al. 2020), the development of selective and efficacious inhibitors remains limited.

In *Caenorhabditis elegans* (*C. elegans*), a nematode commonly used in genetic and developmental research, the CYPs superfamily has been extensively studied because of its functional parallels with human CYP enzymes (Lim et al. 2025). As a genetically tractable model organism with a short lifespan and well-mapped signaling pathways, *C. elegans* provides unique opportunities for high-throughput functional characterization of CYP families (Lim et al. 2023b). The *C. elegans* CYPs are involved in many biological processes including fatty acid synthesis, xenobiotic metabolism, mitochondrial function, lifespan and stress responses (Lim et al. 2022, 2024). These roles mirror many of the biological processes regulated by human CYPs, making nematode CYPs highly relevant for translational research.

Studying the function and regulation of *C. elegans* CYP13 enzymes provides important insights into their biological roles and highlights their potential as models for advancing our understanding of human health (Lim et al. 2022; Deji-Oloruntoba et al. 2025). CYP13 family are involved in xenobiotic metabolism, helping *C. elegans* detoxify harmful environmental compounds, a process analogous to the role of human cytochrome P450 enzymes in drug metabolism. CYP13 family has been linked to responses against oxidative stress, which parallels human CYP enzymes that protect cells from reactive oxygen species and contribute to aging-related processes (Sikder et al. 2025). By studying how these enzymes are regulated such as their induction by stressors, dietary components, or signaling pathways like the nuclear hormone receptor DAF-12 researchers can better understand conserved mechanisms of detoxification, stress resistance, and longevity (Lin et al. 2023; Lim et al. 2024; Teuscher et al. 2024). These parallels make *C. elegans* CYP13 enzymes powerful models for exploring how disruptions in P450 function contribute to human diseases, including cancer, neurodegeneration, and metabolic disorders. This review aims to provide a comprehensive overview of the CYP13 family in *C. elegans*, detailing their expression patterns, functional characteristics and their relevance to human health. Importantly, we will also highlight gaps in current understanding, such as the limited functional characterization of several CYP13 isoforms, incomplete mapping of their regulatory pathways, and the lack of structural data to inform mechanistic comparisons with mammalian orthologs. Addressing these gaps will not only improve our fundamental understanding of P450 biology in *C. elegans* but also guide the development of translational applications, including the use of nematode models for toxicology screening, drug metabolism studies, and aging research.

## *C. elegans* CYP13 family and its human homologues

The *C. elegans* CYP13 family consists of multiple isoforms including *cyp-13A1, cyp-13A2, cyp-13A3, cyp-13A4, cyp-13A5, cyp-13A6, cyp-13A7, cyp-13A8, cyp-13A10, cyp-13A11, cyp-13A12, cyp-13B1* and *cyp-13B2*. Referring to the WormBase database (https://www.alliancegenome.org/members/wormbase), CYP13 enzyme in *C. elegans* are membrane-associated heme-containing NADPH-dependent monooxygenases that catalyze the oxidative metabolism of a variety of exogenous compounds and endogenous substrates. These enzymes are predicted to perform several molecular functions, including heme binding activity, iron ion binding activity, and monooxygenase activity.

**Fig. 1:**
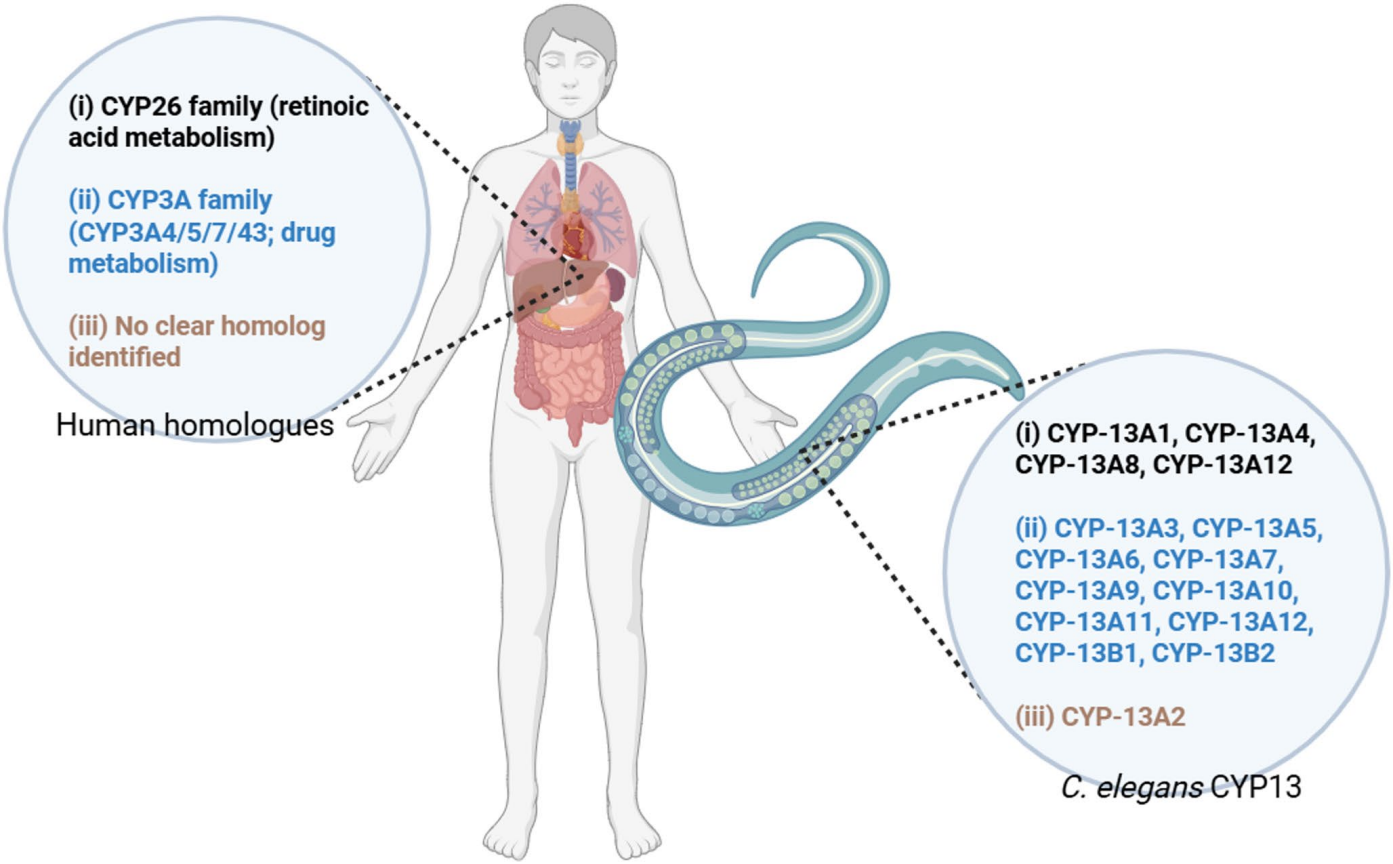
CYP13 isoforms in *C. elegans* and its human homologues

Also based on WormBase, human orthologs of the CYP-13 family are implicated in various disorders such as Ghosal hematodiaphyseal syndrome, cerebral infarction, essential hypertension, familial Mediterranean fever, and leukemia (multiple subtypes). Orthologous genes include TBXAS1 (thromboxane A synthase 1), CYP3A4, CYP3A5, CYP3A43, and CYP3A7–CYP3A51P. Functional studies using large-scale RNAi screens in *C. elegans* have revealed isoform-specific phenotypes: *cyp-13A4*: loss of activity results in uncoordinated locomotion, reduced and/or slow growth, altered adult lifespan, and cadmium hypersensitivity; *cyp-13A5*: knockdown leads to uncoordinated locomotion, decreased/slow growth, and cadmium hypersensitivity; *cyp-13A6*: loss of activity causes slow growth and cadmium hypersensitivity; expression is upregulated by tetrachlorobiphenyl PCB52; *cyp-13A7*: knockdown results in uncoordinated locomotion and slow growth; *cyp-13A7*::*gfp* promoter fusion is induced in the intestine by rifampicin; *cyp-13A8*: loss of function leads to low levels of embryonic lethality, sluggish locomotion, and a clear appearance; and *cyp-13A11*: expression is upregulated following exposure to PCB52. Figure 1 showed the CYP13 isoforms in *C. elegans* and its human homologues.

In a study that aimed to establish *C. elegans* as a model to study the function of vitamin A metabolism, the National Center for Biotechnology Information BLASTP protein database search engine was used to identify *C. elegans* homologs of the known human vitamin A metabolism proteins (Joseph et al. 2021). It was identified that CYP-13A8 is homologous to human CYP26B1, CYP-13A1 and CYP-13A12 are homologous to human CYP26C1, while CYP-13A4 homologous to CYP26C1 (Joseph et al. 2021).

## CYP13 in *C. elegans*

### Lifespan regulation

The amyloid precursor protein (APP) family has long been implicated in Alzheimer’s disease (AD) and aging, but functional redundancy in mammals (APLP1/2) has complicated mechanistic studies (Shariati and De Strooper 2013). *C. elegans*, with its single APP ortholog *apl-1*, offers a tractable model to investigate conserved pathways (Yadav et al. 2025). Notably, APL-1 exerts tissue-specific effects on lifespan: neuronal overexpression shortens lifespan, while hypodermal overexpression promotes longevity through repression of the heterochronic transcription factor LIN-14. This repression preserves youthfulness and requires signaling through DAF-16/FOXO, HSF-1, and DAF-12 (Ewald et al. 2016). Evidence that hypodermal APL-1 modulates LIN-14 activity in aging adults comes from analysis of canonical LIN-14 target genes: *cyp-13, ins-33*, and *C15C7.5* (Ewald et al. 2016). In early larval stages, high LIN-14 levels drive the upregulation of *C15C7.5* while repressing *cyp-13* and *ins-33* (Ewald et al. 2016). During aging, however, hypodermal APL-1 reverses this regulatory program—*cyp-13* and *ins-33* become upregulated, while *C15C7.5* is suppressed (Ewald et al. 2016).

### Apoptotic DNA degradation

A genetic screen utilizing RNAi in *C. elegans* has identified several proteins essential for apoptotic DNA degradation (Kalinowska et al. 2005). Among these were four nucleases encoded by the *cell death-related nucleases (crn)* genes: *crn-1, crn-4, crn-5* together with *cyp-13*, which has been proposed to act in cooperation with the mitochondrial nuclease *CPS-6/EndoG* (Kalinowska et al. 2005). Collectively, these nucleases, together with the apoptosis-related protein *WAH-1*, are thought to assemble into a DNA-degradation complex known as the “degradosome” (Kalinowska et al. 2005). Within this complex, *cyp-13* functions as an endonuclease, highlighting its dual relevance not only in apoptotic DNA clearance but also in broader processes such as lifespan regulation, where its transcriptional modulation links developmental timing factors (e.g., *LIN-14*) to aging pathways.

### Xenobiotic metabolism: toxicity

The *C. elegans cyp-13A6, cyp-13A7, cyp-13A10, cyp-13A3* and *cyp-13A1* were significantly differentially expressed after AFB1 treatment, while *cyp-13A6, cyp-13A9, cyp-13A8, cyp-13A10, cyp-13A7* and *cyp-13A1* were significantly differentially expressed after PCB1254 treatment (Karengera et al. 2022).

### Behavioural response to reoxygenation

The cytochrome P450 enzyme CYP-13A12 has been shown to play a pivotal role in the sustained phase of the O2-ON response in *C. elegans*. This role helps to explain why the *egl-9* mutant phenotype cannot be fully rescued by the *n5590* allele during the initial phase of the response (Ma et al. 2013). Like most, if not all, *C. elegans* CYPs, CYP-13A12 requires the activity of EMB-8, a CYP reductase that transfers electrons to CYP enzymes. The temperature-sensitive mutation *emb-8(hc69)* results in an embryonic lethal phenotype. Importantly, *emb-8(hc69)* mutants raised at permissive temperatures until young adulthood, and subsequently shifted to nonpermissive temperature in combination with *Escherichia coli*-mediated RNAi against *emb-8*, displayed an almost complete abolition of the O2-ON response (Ma et al. 2013). These findings indicate that CYP-13A12 is required specifically for the sustained phase of the response, while additional CYPs likely cooperate with CYP-13A12 to regulate both the initial and sustained phases.

Ma et al. (2013) further identified an EGL-9–HIF-1 pathway that regulates CYP-eicosanoid signaling, demonstrating that polyunsaturated fatty acids (PUFAs) confer a rapid reoxygenation response through CYP-generated eicosanoids. This established a direct mechanistic link among CYP enzymes, PUFA-derived eicosanoids, and animal behavioral responses to reoxygenation. Because the molecular mechanisms underlying O2 and PUFA homeostasis are highly conserved, the O2-ON response in *C. elegans* is considered analogous to the mammalian cellular and tissue responses observed in ischemia–reperfusion injury. Thus, the EGL-9–HIF-1–CYP pathway represents an evolutionarily conserved mechanism controlling responses to reoxygenation after anoxic stress (Ma et al. 2013).

A genetic suppressor screen provided direct evidence for CYP-13A12 as a causal regulator of the O2-ON response, suggesting that CYP-derived eicosanoids act as specific regulators of body wall muscle activity in worms, akin to their role in modulating cardiomyocyte and vascular smooth muscle contractility in mammals (Ma et al. 2013). Among these lipid mediators, the CYP-dependent eicosanoid 17,18-EEQ was identified as a signaling molecule directly involved in modulating the O2-ON behavioral response. Sequence analysis revealed that CYP-13A12 shares 32% amino acid identity with human CYP3A4, a cytochrome P450 enzyme predominantly expressed in the liver but also present in the brain and other extrahepatic tissues, making CYP3A4 its closest human homolog (Keller et al. 2014).

Intriguingly, CYP-13A12 was shown to localize primarily in the marginal cells (MCs) of the *C. elegans* pharynx (Keller et al. 2014). These MCs are enriched in mitochondria, suggesting potential active, non-structural roles beyond their established anatomical functions. However, the mechanism by which CYP-eicosanoids synthesized in the pharynx influence body wall muscle activity remains unclear. One proposed model is that 17,18-EEQ is secreted by the MCs and subsequently detected by nearby sensory neurons, which then activate neural circuits governing forward and backward locomotion, thereby triggering the O2-ON response (Keller et al. 2014).

### Ubiquitylation of nucleosomes on histone H2A

Repression of *cyp* genes represents a conserved function shared by the human tumor-suppressor genes *BRCA1* and *BARD1* and their *C. elegans* homologs *brc-1* and *brd-1*. In human cells, ubiquitylation of histone H2A serves as the signal for *cyp* gene repression, and loss of either *BRCA1* or *BARD1* abolishes this repression (Thapa et al. 2023). A similar mechanism has been demonstrated in *C. elegans*, where loss of repression of *cyp-13A11* and *cyp-13A5* was observed in a CRISPR-generated *brc-1* deletion allele (*gk5332*) and in a *brd-1* mutant allele (*dw1*). Interestingly, the spectrum of affected *cyp* genes differs between the two mutants: *brc-1* mutation additionally de-represses *cyp-13A2* and *cyp-13A10*, which are unaffected by *brd-1* mutation, whereas *brd-1* mutation results in loss of repression of *cyp-13A4*, *cyp-13A6*, *cyp-13A8*, and *cyp-13A12*, which are not affected in *brc-1* mutants (Thapa et al. 2024). BRC-1 and BRD-1 are predicted to mediate gene repression through H2A ubiquitylation. Therefore, the continued repression of many *cyp-13A* genes in the DW102 strain is particularly surprising, given that DW102 animals exhibit loss of ubiquitylated H2A near satellite repeats, a phenotype comparable to that seen in *C. elegans* carrying the *brc-1(ok1261)* allele (Thapa et al. 2024).

## Potential mechanism of action of CYP13

The *cyp-13* family in *Caenorhabditis elegans* represents a multifunctional group of cytochrome P450 enzymes whose activities extend far beyond classical xenobiotic detoxification. Evidence from genetic, biochemical, and behavioral studies indicates that these enzymes act at the crossroads of developmental regulation, apoptosis, metabolism, neuronal signaling, and chromatin control, thereby integrating environmental and genetic inputs into organismal physiology. In the context of aging, *cyp-13* is a transcriptional target of the APP ortholog *apl-1* and its downstream effector *lin-14*. During early larval development, high *lin-14* levels suppress *cyp-13* expression, whereas in adulthood, hypodermal APL-1 represses *lin-14* activity, enabling upregulation of *cyp-13*. This shift aligns with activation of canonical longevity regulators including DAF-16/FOXO, HSF-1, and DAF-12, linking developmental timing cues with metabolic adaptations that promote extended lifespan (Ewald et al. 2016).

Beyond its role in longevity, *cyp-13* has also been implicated in apoptotic DNA degradation. Acting as part of the degradosome complex, it functions in coordination with the mitochondrial nuclease CPS-6/EndoG and the apoptosis-related protein WAH-1. Within this assembly, CYP-13 exhibits endonuclease activity, contributing to the efficient clearance of fragmented DNA during programmed cell death (Kalinowska et al. 2005). This unusual function highlights the dual identity of *cyp-13* as both a metabolic enzyme and a nuclease with roles in genome maintenance. Members of the *cyp-13A* subfamily are further responsive to chemical stress. Exposure to aflatoxin B1 and PCB1254 induces the transcription of multiple isoforms, including *cyp-13A1, cyp-13A3, cyp-13A6, cyp-13A7, cyp-13A8, cyp-13A9,* and *cyp-13A10* (Karengera et al. 2022). This pattern of induction reflects their role in xenobiotic metabolism, where CYP-13 enzymes likely provide protection against toxicants, although—as in mammals—bioactivation of harmful intermediates cannot be excluded.

A particularly well-characterized function of CYP-13 is its role in oxygen sensing and behavioral adaptation to reoxygenation stress. CYP-13A12 is required for the sustained phase of the O2-ON response, a survival behavior triggered upon reoxygenation following hypoxia. This activity depends on electron transfer from the reductase EMB-8 and is regulated by the EGL-9–HIF-1 pathway (Ma et al. 2013). By converting polyunsaturated fatty acids into signaling eicosanoids such as 17,18-EEQ, CYP-13A12 couples oxygen fluctuations with neuronal activity, thereby modulating locomotor behavior. Localization of CYP-13A12 in the mitochondria-rich marginal cells of the pharynx further suggests that it operates at metabolic–neuronal interfaces (Keller et al. 2014). Regulation of *cyp-13* expression also occurs at the epigenetic level. The tumor suppressor homologs *brc-1* and *brd-1* repress distinct subsets of *cyp-13A* genes through ubiquitylation of histone H2A, paralleling the role of BRCA1 and BARD1 in mammals. Mutations in either gene lead to selective de-repression of different *cyp-13A* isoforms, indicating isoform-specific chromatin control. Interestingly, certain strains retain repression of many *cyp-13A* genes despite showing global reductions in H2A ubiquitylation, suggesting the existence of compensatory regulatory mechanisms (Thapa et al. 2024).

**Fig. 2:**
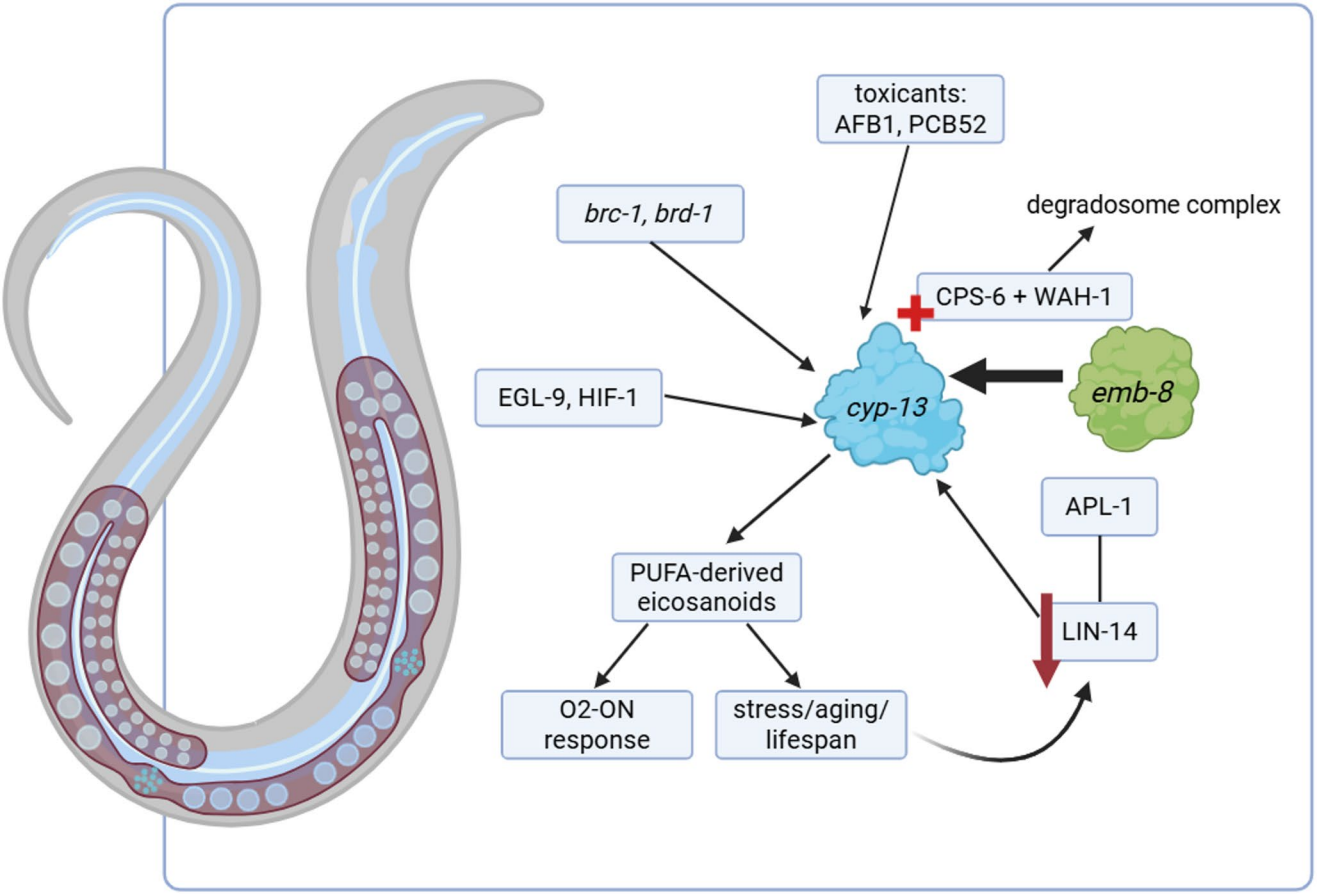
*cyp-13* as a molecular hub connecting biological processes in *C. elegans*. CYP-13 integrates signals from upstream regulators (e.g., LIN-14, EGL-9–HIF-1, BRC-1/BRD-1) and cooperates with effectors such as DAF-16/FOXO, HSF-1, DAF-12, and EMB-8 to mediate diverse physiological outcomes including lifespan regulation, apoptotic DNA degradation, xenobiotic metabolism, oxygen reoxygenation responses, and chromatin-based gene repression

Together, these findings highlight CYP-13 as a multifunctional regulator that connects developmental timing, apoptosis, detoxification, oxygen sensing, and chromatin dynamics as summarised in Table 1. Its activity is shaped by diverse upstream inputs including *apl-1/lin-14*, *egl-9/hif-1*, *emb-8*, and *brc-1/brd-1* and exerts downstream effects on lifespan, genome stability, metabolic defense, and behavioral adaptation. This integration of pathways underscores CYP-13 as a central node in *C. elegans* biology and a valuable model for exploring conserved cytochrome P450 mechanisms. Figure 2 illustrates the proposed mechanisms of CYP-13 action in *C. elegans*, integrating upstream regulators, downstream effectors, and physiological outcomes across lifespan regulation, apoptosis, xenobiotic metabolism, oxygen sensing, and chromatin dynamics.

**Table 1:** Upstream regulators and downstream outcomes of *cyp-13* in *C. elegans*

Biological process	Upstream regulators/effectors	*cyp-13* role/mechanism	Downstream effectors/outcomes	References
Lifespan regulation	*apl-1* (APP ortholog)→represses lin-14→derepresses *cyp-13*; signaling through DAF-16/FOXO, HSF-1, DAF-12	Transcriptional target of APL-1/*lin-14* pathway; expression increases with aging	Lifespan extension; preservation of youth-associated transcriptional programs	Ewald et al. (2016)
Apoptotic DNA degradation	Pro-apoptotic signals; CPS-6/EndoG; WAH-1	Functions as an endonuclease within the degradosome complex	Clearance of apoptotic DNA; maintenance of genomic integrity during programmed cell death	Kalinowska et al. (2005)
Xenobiotic metabolism – toxicity	Environmental toxicants (e.g., AFB1, PCB1254, PCB52, rifampicin)	Multiple *cyp-13A* isoforms induced in intestine and other tissues	Detoxification and/or bioactivation of xenobiotics; survival under chemical stress	Karengera et al. (2022); WormBase
Behavioral response to reoxygenation	*egl-9/hif-1* pathway; *emb-8* (CYP reductase); PUFAs	CYP-13A12 metabolizes PUFAs to generate eicosanoids (e.g., 17,18-EEQ); localized in marginal cells of pharynx	Sustained O₂-ON response; modulation of body wall muscle activity via neuronal circuits	Ma et al. (2013), Keller et al. (2014)
Ubiquitylation of nucleosomes on histone H2A	*brc-1* (BRCA1 homolog) and *brd-1* (BARD1 homolog)	Subset-specific repression of *cyp-13A* isoforms via H2A ubiquitylation	Epigenetic control of *cyp* expression; selective de-repression in mutants	Thapa et al. (2024)

## Conserved functions between *C. elegans* and humans

The study of CYP-13 in *C. elegans* provides valuable insights into conserved cytochrome P450 biology, with clear parallels to human systems. CYP-13A12, the closest homolog of human CYP3A4, regulates the O₂-ON response via eicosanoid signaling, mirroring the role of CYP-derived metabolites in mammalian cardiomyocyte and vascular smooth muscle function. The EGL-9–HIF-1–CYP axis that controls reoxygenation in worms parallels hypoxia–ischemia pathways in mammals, highlighting evolutionary conservation in stress responses (Pender and Horvitz 2018). Similarly, repression of *cyp* genes through histone H2A ubiquitylation by *brc-1* and *brd-1* reflects the gene regulatory functions of BRCA1 and BARD1 in humans, linking P450 regulation to conserved tumor suppressor pathways. Beyond stress responses and epigenetic control, the involvement of *cyp-13* in xenobiotic metabolism underscores its relevance as a model for pharmacological studies, while its integration with APP- and EndoG-related pathways situates it within conserved networks of aging and apoptosis. Despite these parallels, significant gaps remain, including limited structural characterization of CYP-13 isoforms, incomplete mapping of regulatory networks, and insufficient functional validation of their mammalian counterparts. Bridging these gaps will be essential to fully leverage *C. elegans* as a translational model for human P450 biology.

## Future directions and conclusion

Despite significant advances in understanding the function of CYP-13 in *C. elegans*, several limitations remain. Functional characterization has primarily focused on a subset of isoforms such as *cyp-13A1, cyp-13A3, cyp-13A6,* and *cyp-13A12*, while others remain poorly understood. The precise biochemical activities of many CYP-13 enzymes have not been defined, and direct enzymatic assays confirming their predicted substrates are lacking. For example, although *cyp-13A12* is linked to PUFA-derived eicosanoid production, the exact spectrum of metabolites and their receptor targets remain unknown. Similarly, the proposed nuclease activity of *cyp-13* during apoptotic DNA degradation is intriguing but requires biochemical validation, as it diverges from the canonical monooxygenase functions of P450s. Another limitation is the lack of structural data: crystal structures or high-resolution models of CYP-13 isoforms are unavailable, restricting mechanistic insights and comparative studies with human homologs. Finally, many regulatory connections, such as the influence of epigenetic repressors (*brc-1, brd-1*) or developmental timing genes (*lin-14*), have been inferred from gene expression studies but require deeper mechanistic dissection at the molecular and cellular levels.

Future research should prioritize systematic functional characterization of the entire CYP-13 family. This includes biochemical identification of substrates and metabolites, structural studies to define active site architectures, and genetic dissection of isoform-specific roles. High-throughput metabolomic and lipidomic profiling in wild-type and *cyp-13* mutants will be essential to map metabolic pathways mediated by these enzymes. Since several isoforms show inducibility by xenobiotics, further toxicological studies are needed to assess whether CYP-13 enzymes act predominantly in detoxification or bioactivation pathways. Another promising direction is to explore the interplay between CYP-13 activity and epigenetic regulation, particularly how H2A ubiquitylation and chromatin state influence *cyp-13* expression under stress and aging conditions. Cross-species comparative studies should also be expanded, as homologs such as human CYP3A4 and CYP26 isoforms suggest conserved metabolic and regulatory functions. Leveraging CRISPR-based gene editing and single-cell transcriptomics in *C. elegans* will help resolve tissue-specific roles of CYP-13 enzymes, while translational studies could clarify their relevance to human disease models including aging, cancer, and ischemia–reperfusion injury.

The CYP-13 family in *C. elegans* emerges as a multifunctional group of P450 enzymes that bridge developmental, metabolic, and stress-responsive processes. Far from acting solely as xenobiotic metabolizers, CYP-13 isoforms integrate upstream signals from developmental timing genes, hypoxia pathways, mitochondrial reductases, and chromatin regulators to influence outcomes such as lifespan, apoptotic DNA clearance, xenobiotic detoxification, oxygen-sensing behavior, and epigenetic repression. This integrative role positions CYP-13 as a central regulatory hub in nematode physiology and underscores its utility as a model for understanding conserved cytochrome P450 mechanisms in higher organisms. Continued exploration of CYP-13 functions will not only advance our understanding of nematode biology but also provide translational insights into human health and disease, particularly in the areas of drug metabolism, neurodegeneration, stress adaptation, and cancer biology.

## Data Availability

The data that support the findings of this study are available on request from the corresponding author. The data are not publicly available due to privacy or ethical restrictions.
